# Risk of Parkinson’s disease in people aged ≥50 years with new-onset anxiety: a retrospective cohort study in UK primary care

**DOI:** 10.3399/BJGP.2023.0423

**Published:** 2024-06-25

**Authors:** Juan Carlos Bazo-Alvarez, Danielle Nimmons, Kate Walters, Irene Petersen, Anette Schrag

**Affiliations:** Research Department of Primary Care and Population Health, Centre for Ageing and Population Studies, UCL, London.; Research Department of Primary Care and Population Health, Centre for Ageing and Population Studies, UCL, London.; Research Department of Primary Care and Population Health, Centre for Ageing and Population Studies, UCL, London.; Research Department of Primary Care and Population Health, Centre for Ageing and Population Studies, UCL, London.; Department of Neurology, Institute of Neurology, UCL, London.

**Keywords:** anxiety, Parkinson’s disease, incidence, electronic health records, primary health care

## Abstract

**Background:**

A history of anxiety is more common in people with Parkinson’s disease (PD). The prospective risk of PD in those newly presenting with anxiety and factors that increase the risk of PD in patients with anxiety have not been investigated.

**Aim:**

To investigate the incidence of PD in people with anxiety aged ≥50 years and clinical features associated with later diagnosis of PD in people with anxiety.

**Design and setting:**

A retrospective cohort study using UK primary care data between 2008 and 2018, assessing patients with new-onset anxiety aged ≥50 years.

**Method:**

Weibull survival regression models were fitted and hazard ratios (HRs) for modelling time-to-PD was estimated in those with and without anxiety, and when determining the risk of developing PD in those with anxiety. Results were adjusted for sociodemographic and lifestyle factors, and relevant physical and mental health conditions.

**Results:**

The risk of PD increased two-fold compared with the non-anxiety group after adjustment for age, sex, social deprivation, lifestyle factors, severe mental illness, head trauma, and dementia (HR 2.1, 95% confidence interval = 1.9 to 2.4). In those with anxiety, the presence of depression, hypotension, tremor, rigidity, balance impairment, constipation, sleep disturbance, fatigue, and cognitive impairment were associated with an increased risk of developing PD.

**Conclusion:**

The risk of developing PD was at least doubled in people with anxiety compared with those without. The clinical features of those who developed PD can help identify patients presenting with anxiety who are in the prodromal phase of PD.

## Introduction

Anxiety is common in older adults, with 12-month prevalence reported to be around 12%.^1^ Age at onset of anxiety disorders is typically in earlier life and incidence in older age has been reported to be associated with subjective memory complaints as well as subsequent cognitive decline.^2^^–^4

Parkinson’s disease (PD) is the second most common neurodegenerative condition worldwide, and it is estimated that it will affect 14.2 million people by 2040, mostly because of an increase in life expectancy.^5^ Patients with PD can be affected by a range of motor and non-motor symptoms^6^ and may present with non-motor symptoms such as constipation, depression, and olfactory symptoms years before motor symptoms develop.^7^ Presence of anxiety is known to be increased in the prodrome of PD, but the prospective risk of PD in those aged ≥50 years with new-onset anxiety is not known. In addition, it is unclear whether other prodromal features of PD are present in this population that may help improve early recognition and elucidate progression of underlying pathology. One population-based cohort study of 174 776 adults aged <100 years without neurological conditions found that after adjusting for age, sex, medications, and comorbidities, patients with a recorded diagnosis of anxiety or an anxiolytic prescription were more likely to develop PD than those without (hazard ratio [HR] 1.38, confidence interval [CI] = 1.26 to 1.51); and those with more severe anxiety were at greater risk.^8^ However, this study did not take into consideration important factors, such as lifestyle factors or socioeconomic status, and other prodromal features were not examined.

The Health Professionals Follow-Up Study is a cohort study that used the Crown-Crisp Phobic Anxiety index in 35 815 males aged between 40 and 75 years and investigated the incidence of PD over 12 years in those with the highest and lowest levels of anxiety.^9^ There were 189 new cases of PD and after adjusting for age, smoking, and caffeine intake, the relative risk of PD among males with the highest level of anxiety was 1.5 (95% CI = 1.0 to 2.1, *P* = 0.01) compared with males with the lowest level of anxiety. A limitation of this study was that it only focused on male healthcare professionals, who were mostly White.

In addition, among 156 people who developed PD over a period of 40 years in a cohort study of 7216 people aged between 20 and 69 years in Rochester, MN, an anxious personality was associated with an increased risk of PD (HR 1.63, 95% CI = 1.16 to 2.27).^10^

**Table:** How this fits in

Presence of anxiety is known to be increased in the prodrome of Parkinson’s disease (PD). This study investigated the risk of developing PD in people with anxiety compared with those without anxiety, accounting for a number of confounding variables. The results suggest that there is a strong association between anxiety and later diagnosis of PD in patients aged ≥50 years who present with a new diagnosis of anxiety. This provides evidence for anxiety as a prodromal presentation of PD.

None of the above studies examined clinical features that could help predict risk of PD. In addition, variables known to be associated with anxiety such as lifestyle factors, socioeconomic status, or presence of comorbidities such as dementia and head injury were not considered. The aim of this study was therefore to investigate the risk for developing PD in people with anxiety compared with those without anxiety, accounting for a number of confounding variables. Furthermore, the study sought to identify risk factors for later diagnosis of PD in people presenting with anxiety aged >50 years.

## Method

### Data source

The data source was The IQVIA Medical Research Database database, which includes de-identified data from The Health Improvement Network (THIN), a large UK primary care dataset that provides routinely collected electronic health records that are using the In Practice Systems (IPS), also called Vision GP. Data are recorded using Read codes, such as diagnoses and symptoms referrals. THIN data includes about 15.6 million patients, of which 3 million are active patients, from 711 practices whose medical records can be prospectively followed.^11^ There are two main quality markers for these data: a) the acceptable computer usage (ACU), which is reached when a general practice has, on average, at least two therapy records, one medical record, and one additional health data record per patient in a year;^12^ and b) acceptable mortality reporting (AMR), which is reached when mortality records in a general practice are consistent with the official national statistics.^13^ In this study, data were only included from general practices when they met the criteria for ACU and AMR. THIN data broadly represents the UK in terms of demographics, social deprivation, and chronic diseases.^14^

### Cohort identification

Within THIN, all people aged between 50 and 99 years who were registered with a participating practice between 1 January 2008 and 31 December 2018 and had ≥1 anxiety record in the GP database after ≥1 year of no previous records of anxiety were identified. Those aged <50 years were excluded as the population with younger-onset PD may have different characteristics and associations with anxiety.

At the time of first record of anxiety, each person was matched with four other unexposed people based on their sex and age. This selection process ran forward in time, from the initiation until the end of the observation period, setting the matching date for unexposed people as their index date. An incidence and dynamic cohort study was used, where people can initially act as a control for someone else but can eventually become exposed (that is, if they later develop anxiety). This approach is known as exposure density sampling and has been validated elsewhere.^15^ The code list for defining people with cases of anxiety is provided in Supplementary Box S1.

### Outcomes, covariates, and analysis

For the main analysis of comparing the risk of developing PD between people with and without anxiety, Weibull survival regression models were fitted for modelling time-to-event for PD, and HRs were estimated. First, HR estimates were unadjusted, followed by HR estimates adjusted for sociodemographics, that is, sex, age, and social deprivation measured with the Townsend index, from least (one) to most (five) deprived quintile (model 1). The Townsend score was calculated at lower-layer super output areas level using national census data, grouping into quintile based on the deprivation score. The sociodemographics and lifestyle covariates (smoking, alcohol drinking, and body mass index [BMI]) were included in model 2, and sociodemographics, lifestyle covariates, and relevant physical and mental health conditions were included in model 3.

The models were built stepwise to explore the associations accounting for sociodemographic factors only, then the addition of lifestyle factors, and then adding in health conditions that might confound the relationships. All covariates were measured at baseline (that is, date of anxiety record for each exposed person and their matched controls). A directed acyclic graph is shown in Supplementary Figure S1 demonstrating the associations between variables considered as potential confounders in the analysis.^16^ Survival predictions were used for visualising the trajectory of people with and without anxiety (that is, survival figures), for both the unadjusted and model 3 estimates. In these figures, event-free survival (or PD-free survival) is when a person has not presented the outcome at a given time point of follow-up.

To determine which factors contribute to the prediction of PD in people with incident anxiety from the incident and dynamic cohort described above, several risk factors for developing PD were evaluated (that is, risk of developing PD given an incident anxiety diagnosis). The presence of risk and prodromal features of PD at any time point were examined between the anxiety diagnosis date up to 1 year before the date of PD diagnosis. The 1-year interval before diagnosis of PD was chosen to account for diagnostic delays for PD. For each risk factor, the risk of developing PD was determined, unadjusted, and adjusted for the other risk and prodromal features.

For survival models, the proportional hazard assumption was evaluated graphically. Missing data on covariates were handled by multiple imputation with chained equations. Imputation models included one auxiliary variable (that is, systolic blood pressure) and a Nelson–Aalen estimator. Twenty datasets were imputed, specifying 100 iterations for the burn-in period for each chain (one chain per imputation). The survival models were fitted in each of the 20 datasets and their estimates summarised applying Rubin’s rules. Multiple imputation codes are reported in Supplementary Box S2. All estimates were given with 95% CIs using robust standard errors to consider clustering by practice.

The statistical analyses were performed using Stata/MP (version 16.1) for Windows.

## RESULTS

### Comparing the risk of developing PD in people with and without anxiety

There were 38 510 males and 70 925 females who were diagnosed with a first episode of anxiety; and 324 670 males and 553 586 females in the unexposed group. In the anxiety group, sleep problems, depression, fatigue, and constipation were prodromal features that were more common (Table 1).

**Table 1.** Characteristics of people with and without anxiety (*N* = 987 691)

**Characteristic**	**Non-anxiety cohort (*n* = 878 256)**		**Anxiety cohort (*n* = 109 435)**	

	***n* ^a^**	%**^a^**	***n* ^a^**	%**^a^**
**Sex**				
Male	324 670	36.97	38 510	35.19
Female	553 586	63.03	70 925	64.81

**Age, years**				
50–54	343 552	39.12	41 794	38.19
55–59	129 533	14.75	16 288	14.88
60–64	121 250	13.81	14 869	13.59
65–69	89 183	10.15	11 056	10.10
70–74	75 715	8.62	9724	8.89
75–79	59 062	6.72	7656	7.00
80–84	36 504	4.16	4868	4.45
85–89	18 298	2.08	2505	2.29
90–94	4481	0.51	603	0.55
95–99	678	0.08	72	0.07

**Deprivation quintile**				
1 (least deprived)	242 499	27.61	28 260	25.82
2	214 063	24.37	25 610	23.40
3	184 065	20.96	22 803	20.84
4	143 140	16.30	19 225	17.57
5 (most deprived)	94 489	10.76	13 537	12.37

**Alcohol^b^**				
Non-drinker	372 292	42.39	53 538	48.92
Ex-drinker	175 477	19.98	28 113	25.69
Normal drinker	55 944	6.37	9539	8.72
Heavy drinker	26 271	2.99	5209	4.76
Very heavy drinker	864	0.10	183	0.17
Missing	247 408	28.17	12 853	11.74

**Smoking**				
Never smoker	372 370	42.40	53 540	48.92
Ex-smoker	182 842	20.82	29 382	26.85
Current smoker	128 077	14.58	22 808	20.84
Missing	194 967	22.20	3705	3.39

**BMI**				
Mean (SD)	27.94 (5.61)	—	27.73 (5.72)	—
Missing	280 128	31.90	13 868	12.71

**Cognitive impairment**	16 578	1.89	3369	3.10

**Erectile dysfunction**	18 767	2.14	2152	2.00

**Sleep problems**	29 989	3.41	8813	8.10

**Balance impairment**	3365	0.38	606	0.55

**Constipation**	42 838	4.88	9251	8.45

**Depression**	24 820	2.83	9900	9.05

**Dizziness**	40 392	4.60	6464	5.91

**Fatigue**	51 740	5.89	12 330	11.27

**Hypotension**	2877	0.33	393	0.36

**Rigidity**	978	0.11	140	0.13

**Shoulder pain**	43 297	4.93	4002	3.66

**Tremor**	3445	0.39	703	0.64

**Urinary dysfunction**	3006	0.34	468	0.43

a *Unless otherwise stated.*

b *Normal drinker = females <14 units, males <22 units; heavy drinker = females 14–35 units, males 22–49 units; and very heavy drinker = females >35 units, males >49 units. Units are per week. BMI = body mass index. SD = standard deviation.*

Of those who had anxiety, 331 developed PD during the follow-up period. The median time to diagnosis of PD after the first recorded episode of anxiety was 4.9 years (interquartile range 4.0–9.6). The incidence of PD in those with and without anxiety was 1.02 (95% CI = 0.92 to 1.13) and 0.49 (95% CI = 0.47 to 0.52) per 1000 person–years, respectively (Table 2).

**Table 2.** Incidence of Parkinson’s disease in patients with and without anxiety (*N* = 987 691)^a^

**Cohort**	**Incidence per 1000 person–years (95% CI)**	**Unadjusted HR (95% CI)**	**Model 1, HR (95% CI)**	**Model 2, HR (95% CI)**	**Model 3, HR (95% CI)**
**Non-anxiety**	0.49 (0.47 to 0.52)	Reference	Reference	Reference	Reference
**Anxiety**	1.02 (0.92 to 1.13)	2.5 (2.3 to 2.8)	2.7 (2.4 to 2.9)	2.7 (2.4 to 3.0)	2.1 (1.9 to 2.4)

a *Model 1 is a Weibull survival model adjusted for sex, age, deprivation, and for clustering by practice using robust standard errors. Model 2 is a Weibull survival model adjusted for sex, age, deprivation, alcohol, smoking, BMI, and for clustering by practice using robust standard errors. Model 3 is a Weibull survival model adjusted for sex, age, deprivation, alcohol, smoking, BMI, SMI, head trauma, dementia, and for clustering by practice using robust standard errors. BMI = body mass index. HR = hazard ratio. SMI = severe mental illness.*

**Figure 1.**
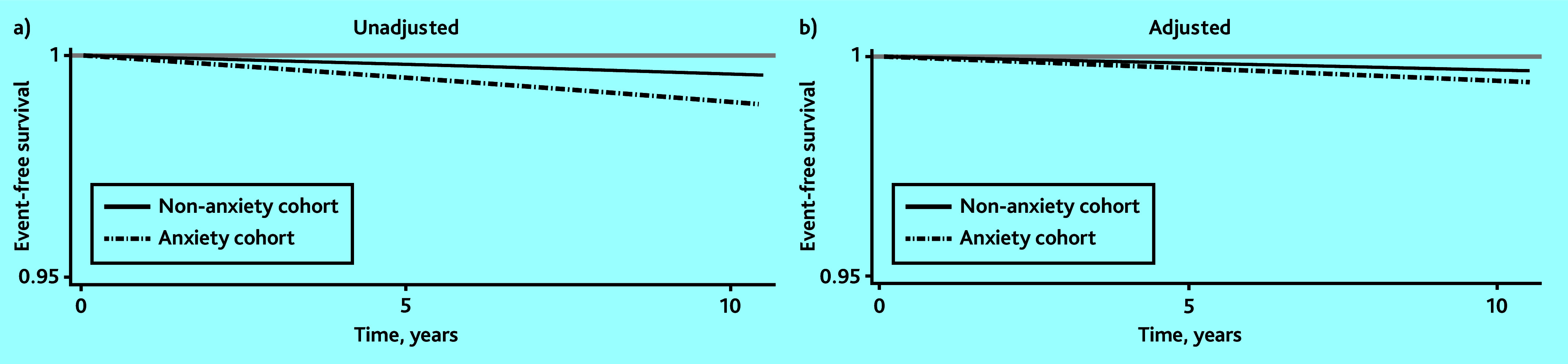
Predicted Parkinson’s disease-free survival for people with and without anxiety: a) unadjusted; and b) adjusted for sex, age, deprivation, alcohol smoking, body mass index, cognitive impairment, erectile dysfunction, sleep problems, balance impairment, constipation, depression, dizziness, fatigue, hypotension, rigidity, shoulder pain, tremor, urinary dysfunction, and for clustering by practice (using robust standard errors).

The risk of PD in people with anxiety was twice the risk of people without, even after adjustment for age, sex, social deprivation, lifestyle factors, severe mental illness, head trauma, and dementia (HR 2.1, 95% CI = 1.9 to 2.4) (Table 2). Also, people without anxiety survived for a longer period without developing a PD event compared with those with anxiety (Figure 1).

The absolute risk of having PD per age band for those with and without anxiety is also reported (see Supplementary Table S1). This may be helpful for clinicians having discussions with their patients about their risk of PD, considering their specific age group.

### Identifying risk factors for developing PD in people with anxiety

Females (HR 0.41, 95% CI = 0.34 to 0.50) and those in the most deprived socioeconomic group (HR 0.60, 95% CI = 0.40 to 0.91) were less likely to be diagnosed with PD (Table 3). When lifestyle factors were included in the model (smoking, alcohol use, and BMI), they were not found to be associated with developing PD.

The following symptoms were associated with developing PD in people with anxiety: depression (HR 1.7, 95% CI = 1.1 to 2.5), sleep disturbance (HR 2.2, 95% CI = 1.5 to 3.2), fatigue (HR 1.8, 95% CI = 1.3 to 2.6), cognitive impairment (HR 1.8, 95% CI = 1.1 to 3.1), hypotension (HR 4.0, 95% CI = 1.7 to 9.7), tremor (HR 21.3, 95% CI = 14.4 to 31.5), rigidity (HR 5.1, 95% CI = 1.2 to 21.2), balance impairment (HR 4.2, 95% CI = 2.1 to 8.3), constipation (HR 2.6, 95% CI = 1.9 to 3.6), but not shoulder pain, dizziness, erectile dysfunction, and urinary dysfunction (Table 3).

**Table 3.** Parkinson’s disease risk factors in people with anxiety (*N* = 109 435)^a^

**Variable**	**Model 1 HR (95% CI)**	**Model 2 HR (95% CI)**	**Model 3 HR (95% CI)**
**Sex**			
Male	Reference	Reference	Reference
Female	0.42 (0.35 to 0.52)	0.40 (0.33 to 0.49)	0.41 (0.34 to 0.50)

**Age, years**			
50–54	Reference	Reference	Reference
55–59	3.4 (2.1 to 5.4)	3.3 (2.1 to 5.3)	3.2 (2.0 to 5.2)
60–64	4.4 (2.9 to 6.9)	4.4 (2.8 to 6.8)	4.0 (2.6 to 6.3)
65–69	8.1 (5.3 to 12.6)	7.9 (5.1 to 12.2)	7.0 (4.5 to 11.1)
70–74	10.2 (6.7 to 15.7)	9.7 (6.3 to 14.9)	8.0 (5.1 to 12.4)
75–79	8.5 (5.4 to 13.5)	7.9 (5.0 to 12.5)	6.5 (4.0 to 10.6)
80–84	8.3 (4.9 to 14.1)	7.5 (4.4 to 12.9)	6.7 (3.8 to 11.6)
85–89	4.4 (1.8 to 10.6)	3.8 (1.6 to 9.4)	3.4 (1.4 to 8.3)
90–94	11.8 (3.5 to 39.0)	9.9 (3.0 to 32.9)	9.8 (2.9 to 32.8)
95–99	0 (0 to 0)	0 (0 to 0)	0 (0 to 0)

**Deprivation quintile**			
1 (least deprived)	Reference	Reference	Reference
2	0.89 (0.66 to 1.2)	0.91 (0.68 to 1.2)	0.91 (0.68 to 1.2)
3	1.1 (0.82 to 1.4)	1.1 (0.86 to 1.5)	1.1 (0.86 to 1.5)
4	0.89 (0.66 to 1.2)	0.98 (0.72 to 1.3)	0.97 (0.72 to 1.3)
5 (most deprived)	0.54 (0.36 to 0.81)	0.61 (0.40 to 0.92)	0.60 (0.40 to 0.91)

**Alcohol^b^**			
Non-drinker	—	Reference	Reference
Ex-drinker	—	0.73 (0.21 to 2.5)	0.73 (0.20 to 2.6)
Normal drinker	—	0.77 (0.17 to 3.5)	0.77 (0.16 to 3.7)
Heavy drinker	—	0.68 (0.15 to 3.2)	0.64 (0.13 to 3.2)
Very heavy drinker	—	0 (0 to 9.1)	0 (0 to 28.2)

**Smoking**			
Never smoker	—	Reference	Reference
Ex-smoker	—	0.99 (0.30 to 3.4)	0.99 (0.27 to 3.6)
Current smoker	—	0.63 (0.15 to 2.7)	0.63 (0.14 to 2.9)

**BMI**	—	0.99 (0.97 to 1.0)	0.99 (0.97 to 1.0)

**Cognitive impairment**	—	—	1.8 (1.1 to 3.1)

**Erectile dysfunction**	—	—	0.96 (0.42 to 2.2)

**Sleep problems**	—	—	2.2 (1.5 to 3.2)

**Balance impairment**	—	—	4.2 (2.1 to 8.3)

**Constipation**	—	—	2.6 (1.9 to 3.6)

**Depression**	—	—	1.7 (1.1 to 2.5)

**Dizziness**	—	—	0.56 (0.29 to 1.1)

**Fatigue**	—	—	1.8 (1.3 to 2.6)

**Hypotension**	—	—	4.0 (1.7 to 9.7)

**Rigidity**	—	—	5.1 (1.2 to 21.2)

**Shoulder pain**	—	—	0.86 (0.41 to 1.8)

**Tremor**	—	—	21.3 (14.4 to 31.5)

**Urinary dysfunction**	—	—	1.8 (0.44 to 7.5)

a *The authors performed multiple imputation (20 datasets) for bias correction. Model 1 is a Weibull survival model adjusted for sex, age, deprivation, and for clustering by practice using robust standard errors. Model 2 is a Weibull survival model adjusted for sex, age, deprivation, alcohol, smoking, BMI, and for clustering by practice using robust standard errors. Model 3 is a Weibull survival model adjusted for sex, age, deprivation, alcohol, smoking, BMI, cognitive impairment, erectile dysfunction, sleep problems, balance impairment, constipation, depression, dizziness, fatigue, hypotension, rigidity, shoulder pain, tremor, urinary dysfunction, and for clustering by practice using robust standard errors.*

b *Normal drinker = females <14 units, males <22 units; heavy drinker = females 14–35 units, males 22–49 units; and very heavy drinker = females >35 units, males >49 units. Units are per week. BMI = body mass index. HR = hazard ratio.*

## Discussion

### Summary

The results suggest that there is a strong association between anxiety and later diagnosis of PD in patients aged >50 years who present with a new diagnosis of anxiety. In people with anxiety, the study confirmed that depression, sleep disturbance, fatigue, cognitive impairment, hypotension, tremor, rigidity, balance impairment, and constipation are risk factors for developing PD.

### Strengths and limitations

This study used a large electronic health record database that is broadly representative of the UK population. Important factors such as socioeconomic status, lifestyle factors, and confounding conditions, such as severe mental illness, were also included and these results therefore add to what is already known in this area. However, the results are limited by the information entered in the medical records by healthcare professionals, which is primarily added for clinical purposes and not research. Mental health is under-recorded in electronic health records including in older people,^17^^,^^18^ in part because of stigma and negative media portrayal.^19^ It is therefore possible that the true impact of anxiety on subsequent PD incidence is therefore not fully captured in these results because of under-reporting and under-recording.

### Comparison with existing literature

The current findings are in line with results from previous studies reporting an increased risk of PD in people with previous anxiety (HR 1.38, 95% CI = 1.26 to 1.51; HR 1.5, 95% CI = 1.0 to 2.1; and HR 1.63, 95% CI = 1.16 to 2.27)^8^^–^10 in a population that is broadly representative of the UK population, even when adjusting for socioeconomic factors, lifestyle factors, and confounding conditions, such as severe mental illness, head trauma, and dementia. It was important to include these conditions as they have been shown to be associated with both anxiety and PD.^20^^–^31 Of note, smoking has consistently been negatively associated with PD, whereas it has been positively associated with anxiety. In this study in patients with anxiety, smoking status was not associated with PD when alcohol use and BMI was also accounted for.

In keeping with PD overall, older people, those in a less deprived socioeconomic group, and male individuals with anxiety were at greater risk of a diagnosis of PD. This is similar to the general population, as incidence of a PD diagnosis is known to be higher in older people, males, and those from higher socioeconomic groups.^32^^,^^33^ Anxiety, on the other hand, is more common in females and tends to start earlier in life.^34^ Primary care doctors under-record anxiety symptoms and diagnoses, sometimes because they do not want to give patients a mental health label.^18^ It is therefore possible that only more severe forms of anxiety were recorded.

The pattern of clinical features associated with later PD was similar to that reported in the general population previously, including motor features (tremor, rigidity, and balance impairment), autonomic features (constipation and hypotension), sleep disturbance, cognitive impairment, and fatigue.^35^^,^^36^ However, the previously reported features that were not associated with an increased risk of PD in the current study were erectile dysfunction, urinary dysfunction, dizziness, and shoulder pain. This may be owing to these symptoms masking an association of specific presentations such as postural hypotension with later diagnosis of PD or owing to under-reporting of these symptoms in general practice. However, as they were previously shown to be associated with later diagnosis of PD in a similar study design,^35^ it may also suggest that patients with PD who present with initial anxiety have a specific clinical phenotype with fewer autonomic features. It has been suggested that PD can begin with different phenotypes, reflecting differences in progression pathways.

The condition has been divided using subtyping systems, including according to age-at-onset categories, motor phenotypes, and by non-motor symptoms,^37^ for example, the neuropsychiatric subtype, where defining symptoms are anxiety and depression, postural instability, and gait disturbances.^38^^,^^39^ There are also subtypes according to whether symptoms do or do not start in the brain (brain- and body-first subtypes).^40^ There is increasing evidence for specific non-motor dominant PD phenotypes and subtyping may help guide research and clinical practice, although this may be challenging as PD is highly heterogeneous and subtypes are likely to overlap.^41^ One specific hypothesis suggests that there is a ‘brain-first’ subtype of PD that starts in the brain and spreads to the peripheral autonomic nervous system, and a ‘body-first’ PD pathology that starts in the enteric or peripheral autonomic nervous system and spreads to the brain,^40^ leading to presentations with orthostatic hypotension, constipation, and olfactory symptoms.^42^ Onset of PD with anxiety as first presentation may fit into this model of the ‘brain-first’ subtype, with less involvement of peripheral autonomic structures and greater involvement of serotonergic structures involved in early stages.^43^ Other features of PD that are thought to be related to serotonergic deficits include fatigue, poor sleep, and depression,^44^ which the study also found to be associated with an increased risk of later PD.

### Implications for research and practice

Anxiety is not as well researched as other prodromal features of PD, such as depression. Further research should explore anxiety in relation to other prodromal symptoms and how this symptom complex is associated with the incidence of PD. This may lead to earlier diagnosis and better management of PD. It could also explore if incidence is affected by severity of anxiety, and the authors of the current study recommend that further studies are needed to explore why there is an increased risk of PD in people with anxiety aged ≥50 years.

In conclusion, there was a two-fold increase in risk of PD in patients with first presentation of anxiety aged >50 years. The clinical features of those who developed PD can help identify patients presenting with anxiety who are in the prodromal phase of PD.

## Data

The Health Improvement Network license prohibits public use of study data and is therefore unavailable.
